# Supplementary material for: An MRI Atlas of Regional Brain Vulnerability to Metastatic Disease
Source: medRxiv. 2026 Jul 1:2026.06.29.26356888. Preprint. [Version 1] doi: 10.64898/2026.06.29.26356888 (PMC13345565; doi:10.64898/2026.06.29.26356888)
Supplement: Supplementary file 2 [file media-2.docx]

**Supplemental Digital Content 2. Figure.** Regional tropism analysis adjusted for regional cerebral blood flow. Repeating the permutation analysis using a normative cerebral blood flow atlas yielded results consistent with the primary analysis, with the same regions remaining significantly over- and underrepresented.
