# Supplementary material for: An MRI Atlas of Regional Brain Vulnerability to Metastatic Disease
Source: medRxiv. 2026 Jul 1:2026.06.29.26356888. Preprint. [Version 1] doi: 10.64898/2026.06.29.26356888 (PMC13345565; doi:10.64898/2026.06.29.26356888)
Supplement: Supplementary file 3 [file media-3.docx]

**Supplemental Digital Content 3. Figure.** Observed versus expected infratentorial metastasis frequency by primary tumor type. Gastrointestinal, breast, genitourinary, and lung carcinomas demonstrated greater-than-expected infratentorial involvement, whereas melanoma did not.
