# Supplementary material for: An MRI Atlas of Regional Brain Vulnerability to Metastatic Disease
Source: medRxiv. 2026 Jul 1:2026.06.29.26356888. Preprint. [Version 1] doi: 10.64898/2026.06.29.26356888 (PMC13345565; doi:10.64898/2026.06.29.26356888)
Supplement: Supplementary file 4 [file media-4.docx]

**Supplemental Digital Content 3. Figure.** Lung vs Melanoma Tumor Locations. **(A)** Patient-level analysis showing the proportion of patients with at least one lesion in each specified compartment, vascular territory, and anatomical lobe. **(B)** Lesion-level analysis showing the proportion of total tumor centroids located within each region. **(C)** Mean tumor mass for lung and melanoma BM patients. Error bars indicate standard deviation. Across metrics, lung metastases demonstrate a significant spatial preference for cerebellar and vertebrobasilar regions relative to melanoma.
